# Synergistic remediation of continuous cropping obstacles in facility agriculture: insights from the *Stropharia rugosoannulata*-Ornamental Sunflower Rotation System

**DOI:** 10.3389/fmicb.2025.1671484

**Published:** 2025-11-07

**Authors:** Jia Lu, Yan Chen, Jing Yan, Huiqing Ye, Xuebin Ying, Xiaohua Zhou, Fangjie Yao, Zufa Zhou

**Affiliations:** 1 Hangzhou Academy of Agricultural Sciences, Hangzhou, China; 2 Engineering Research Center of Ministry of Education of China for Food and Medicine, Jilin Agricultural University, Changchun, China; 3 Hangzhou Rural Revitalization Service Center, Hangzhou, China; 4 Lin′an Agriculture and Forestry Technology Extension Center, Hangzhou, China; 5 Tonglu Country Agricultural Technology Promotion Center, Hangzhou, China

**Keywords:** continuous cropping obstacles, spent mushroom substrate, microbial co-occurrence networks, enzyme stoichiometry, *Stropharia rugosoannulata*, soil microecological restoration

## Abstract

Continuous cropping in facility agriculture induces severe soil degradation through acidification, nutrient imbalance, and pathogen accumulation, posing a significant threat to agricultural sustainability; to address this challenge, we developed an innovative *Stropharia rugosoannulata*-Ornamental Sunflower Rotation System (SR-OS2) incorporating spent mushroom substrate (SMS) and investigated its remediation mechanisms through integrated approaches including soil physicochemical analysis, extracellular enzyme assays, high-throughput sequencing (16S/ITS), co-occurrence network analysis, and Partial Least Squares Path Modeling (PLS-PM). The SR-OS2 system significantly enhanced soil properties by increasing pH (+0.57 units), decreasing electrical conductivity (−37.56%), and boosting available phosphorus (+84.2%), while also shifting microbial communities toward bacterial dominance with a 37.4% increase in bacterial Chao1 diversity and a 39.1% decrease in fungal diversity, alongside strengthened bacterial connectivity (+42%) and reduced fungal modularity in co-occurrence networks. Enzyme stoichiometry further revealed alleviated nitrogen limitation (vector angle: 27.2°–30.9°), and PLS-PM identified dual remediation pathways—a dominant biological pathway (*β* = 0.92) and a physicochemical pathway (*β* = −0.501); these improvements collectively demonstrate that the SR-OS2 system synergistically restores soil microecological functions, providing a sustainable paradigm for agricultural waste valorization and effective management of continuous cropping obstacles.

## 1 Introduction

Continuous cropping obstacles in facility agriculture pose a severe threat to global agricultural sustainability. These obstacles manifest primarily as the deterioration of soil physicochemical properties (acidification, nutrient imbalance) and ecological functional degradation driven by the enrichment of soil-borne pathogens and microbial community dysbiosis, ultimately leading to persistent crop yield reduction (Li Y. et al., 2022). Ornamental sunflower (*Helianthus annuus* L.), an economically and ecologically valuable crop, faces industrial development constraints due to soil health decline induced by continuous cropping (Puttha et al., 2023). Recent studies demonstrate that incorporating spent mushroom substrate (SMS) into soil is an effective strategy for rehabilitating degraded soils. Its mechanisms involve not only enhancing soil organic matter and activating mineral nutrients (Li et al., 2020) but also significantly altering soil microbial community structure and enzyme activity (Gong et al., 2018; Hao et al., 2024). Specifically, SMS from *Stropharia rugosoannulata* cultivation—rich in lignin-degrading enzymes (e.g., lignin peroxidase, LiP) and humus precursors (Hao et al., 2024)—exhibits unique potential for mitigating continuous cropping obstacles. To leverage this potential, we innovatively designed the “*Stropharia rugosoannulata*-Ornamental Sunflower” rotation system (SR-OS2). This system integrates *in-situ* SMS incorporation with coordinated stubble management to simultaneously achieve agricultural waste valorization and soil health restoration.

Although *S. rugosoannulata* SMS input has been confirmed to increase soil organic carbon (SOC) and total nitrogen (TN) pools (Gong et al., 2018), critical knowledge gaps remain regarding its deep-layer mechanisms for rehabilitating continuously cropped soils: (1) The regulatory patterns of SMS input and rotation on cross-seasonal C-N-P stoichiometric dynamics—particularly enzyme activity ratios and vector traits—have not yet been quantified (Hu et al., 2020); (2) Microbial community restructuring (e.g., increased bacterial dominance) (Hernandez et al., 2021) has not been effectively linked to topological evolution in microbial co-occurrence networks (e.g., connectivity, modularity); (3) Integrated empirical models are lacking for SMS-driven “physicochemical-biological” multi-level cascade pathways (e.g., trace element activation → microbial community restructuring → enzyme activity response) (Jansson and Baker, 2016).

To address these gaps, this study established a four-stage SR-OS2 treatment using soil from a 5-year continuously cropped sunflower facility: B1: Untreated soil (control), B2: SMS incorporation, B3: First sunflower crop, B4: Second no-till sunflower crop. Prior to the experiment, this facility had documented issues of stunted plant growth, leaf chlorosis, and an estimated yield reduction of over 20% in the most recent cropping season—symptoms characteristic of severe continuous cropping obstacles.

By integrating soil physicochemical analysis, extracellular enzyme activity assays, and high-throughput sequencing, we aimed to: (1) Decipher C-N-P stoichiometric dynamics: Quantify changes in SOC/TN/AP (available phosphorus) content, enzyme activity ratios, and enzymatic traits; (2) Uncover microbial co-occurrence network restructuring: Analyze topological properties (connectivity, modularity) of bacterial/fungal communities and their coupling with key extracellular enzyme activities; (3) Validate multi-level cascade pathways: Test physicochemical (trace elements → metalloenzyme activation) and biological (SOC/TN → microbial community → enzyme balance) cascading effects. This study provides new insights into the ecological mechanisms underpinning continuous cropping obstacle mitigation and supports the optimization of agricultural waste recycling technologies.

## 2 Materials and methods

### 2.1 Study region and experimental design

Field experiments were conducted from March 2023 to July 2024 in Lin’an District, Hangzhou, Zhejiang Province, China (119°61′E, 30°20′N), a region characterized by a mid-subtropical monsoon climate with a mean annual temperature of 15.4 °C and annual precipitation of 1,000–1,200 mm. The soil, classified as yellow-brown, had undergone 5 years of continuous ornamental sunflower (*Helianthus annuus* L.) cultivation, with initial properties of pH 6.82, EC 120 μS cm^−1^, and SOC 1.25%. We established the SR-OS2 with four treatments: (1) B1: Untreated fallow soil; (2) B2: Soil amended with *S. rugosoannulata* spent mushroom substrate (SMS) incorporated into the 0–20 cm layer after mushroom harvest. SMS was applied at a rate of 10 t/ha. The SMS had the following basic properties: total organic carbon (TOC) = 35.2%, total nitrogen (TN) = 1.8%, and a C/N ratio of 19.6; (3) B3: Sunflower (*H. annuus* cv. ‘Jincancan’) planted post-SMS amendment at 6 × 10^4^ plants ha^−1^ density (90-day growth period); (4) B4: Second sunflower crop planted under no-till conditions after B3 harvest (85-day growth period). A randomized block design with three replicates was implemented, with individual plots (30 m × 20 m) separated by ≥30 m buffers to mitigate edge effects. No additional fertilization was applied during sunflower cultivation. Standard field management practices included drip irrigation to maintain soil moisture at 60–70% of field capacity and manual weeding as necessary.

### 2.2 Soil sampling and pretreatment

Composite soil samples (0–20 cm depth) were collected at critical stages: B1 (initial soil), B2 (30 days post-SMS incorporation), and B3/B4 (immediately after sunflower harvest). Five subsamples per plot were obtained along an “S”-shaped transect within 1 m × 1 m quadrats and homogenized. Samples were partitioned for: (1) fresh storage (4 °C, enzyme assays within 24 h); (2) flash-freezing in liquid N₂ (−80 °C storage for DNA extraction); (3) air-drying (sieved to 2 mm for physicochemical analysis or 0.15 mm for micronutrient detection).

### 2.3 Soil physicochemical analysis

Soil pH and EC were determined in 1:2.5 (w/v) soil-water slurry using a PHS-25 pH meter (Leici, Shanghai) and a DDS-307 conductivity meter (Yidian, Shanghai, China), respectively. SOC was quantified via potassium dichromate oxidation (Keming Bio, Suzhou, China), and TN followed the Kjeldahl method (GB 7173–87; Kjeltec 8,400, Foss, Denmark). AP was extracted with 0.5 M NaHCO₃ (pH 8.5) and measured spectrophotometrically (UV-1800, Shimadzu (Shanghai) Global Laboratory Consumables Co., Ltd., Shanghai, China) using the molybdenum blue method. AK (Available Potassium) was extracted with 1 M NH₄OAc and quantified by flame photometry (FP6410, Jingke, Shanghai). Micronutrients (Ca, Mg, Fe, Mn, Cu, Zn, S) were digested with HNO₃-H₂O₂-HF (170 °C, 4 h) and analyzed by ICP-MS (NexION® 1,000, PerkinElmer), with spike recovery rates of 85–110%.

### 2.4 Extracellular enzyme activity assays

Five extracellular enzymes were assayed colorimetrically: *β*-glucosidase (BG; p-nitrophenyl-β-D-glucopyranoside substrate, 400 nm) for C mineralization; N-acetylglucosaminidase (NAG; p-nitrophenyl-N-acetyl-β-D-glucosaminide, 400 nm) for fungal N cycling; leucine aminopeptidase (LAP; L-leucine-p-nitroanilide, 405 nm) for bacterial N cycling; alkaline phosphatase (ALP; p-nitrophenyl phosphate, 405 nm) for P mineralization; and polyphenol oxidase (PPO; pyrogallol, 430 nm) for lignin degradation. Fresh soil (0.5 g dry-weight equivalent) was incubated with substrate and modified universal buffer (pH 7.0) at 37 °C for 4 h (BG, NAG, LAP, ALP) or 24 h (PPO). Activities are expressed as μmol product g^−1^ soil d^−1^.

### 2.5 Enzyme stoichiometry analysis

Microbial nutrient limitations were evaluated using enzyme vector analysis. Vector length $(L=\sqrt{{\left[\ln(\mathrm{BG})\right]}^{2}+{\left[\ln(\mathrm{NAG}+\mathrm{LAP})\right]}^{2}}),$ indicated carbon limitation (higher values), while vector angle (A = arctan [ln(NAG+LAP)/ln(BG)]), identified nitrogen (<45°) or phosphorus (>45°) limitation. Additional ratios included: BG:(NAG + LAP) (C vs. N acquisition investment), NAG:LAP (fungal vs. bacterial N cycling dominance; >1 = fungal), BG:PPO (labile vs. recalcitrant C decomposition), and BG:ALP (C vs. P acquisition strategy).

### 2.6 Microbial high-throughput sequencing

Soil DNA was extracted using the TIANamp Soil DNA Kit (TIANGEN, Beijing, China). Bacterial 16S rRNA V3–V4 regions were amplified with primers 341F (5’-CCTACGGGNGGCWGCAG-3′)/805R (5’-GACTACHVGGGTATCTAATCC-3′), and fungal ITS1 regions with ITS1F (5’-CTTGGTCATTTAGAGGAAGTAA-3′)/ITS2R (5’-GCTGCGTTCTTCATCGATGC-3′). PCR conditions included: 98 °C for 30 s; 27 cycles of 98 °C for 10 s, 50 °C for 30 s, and 72 °C for 30 s; and final extension at 72 °C for 5 min. Libraries were validated (Agilent 2100 Bioanalyzer, California, USA) and sequenced on the Illumina NovaSeq 6000 platform (PE250; 60,000 reads/sample target depth).

### 2.7 Data analysis

#### 2.7.1 Amplicon sequencing data analysis

Raw sequencing data underwent processing with DADA2 (v1.26) for bacterial 16S rRNA amplicons and UNOISE3 (v11) for fungal ITS sequences to generate amplicon sequence variants (ASVs). Taxonomic assignment was performed against the SILVA v138 database for bacteria and UNITE v8.0 for fungi. Alpha diversity was evaluated using Chao1 richness and Simpson’s evenness indices. FUNGuild (v1.2) was used to predict the ecological functions of the fungal communities. Fungal ASVs were assigned to trophic modes and guilds, and those assigned as ‘highly probable’ or ‘probable’ were retained for subsequent analysis. Linear Discriminant Analysis Effect Size (LEfSe) was performed using the online tool^1^, with an LDA score threshold set to > 3.0 and a significance level of *p* < 0.05 (Kruskal-Wallis test).

#### 2.7.2 Statistical and multivariate analysis

Treatment effects on soil properties, enzyme activities, and diversity metrics were assessed through one-way ANOVA with Fisher’s LSD *post hoc* tests (*p* < 0.05) in R v4.3.2. Microbial community structure variations were visualized via principal coordinates analysis (PCoA) based on Bray-Curtis distances and statistically validated with PERMANOVA (999 permutations; vegan package). Microbial co-occurrence networks were constructed using the SpiecEasi package (v1.1.2) with the MB method (Matrix Bootstrap) for robust association inference. Networks were constructed for each treatment group using all samples within that group. Only robust interactions with |SparCC *ρ*| > 0.6 and FDR-corrected *p* < 0.01 were retained. Network topology metrics (nodes, edges, average degree, transitivity) were computed with `igraph` (v1.6.0). Partial Least Squares Path Modeling (PLS-PM) was implemented via the plspm package (v0.4.9) incorporating six latent variables: (1) Rotation treatment (categorical: B1-B4), (2) Soil properties (observed: SOC, TN, AP, pH), (3) Micronutrients (observed: Mg, Fe, Mn, Cu, Zn), (4) Microbial community (observed: bacterial/fungal Chao1 and Simpson indices), (5) Enzyme ratios (observed: BG:(NAG+LAP), BG:PPO, BG:ALP, NAG:LAP), and (6) Enzyme activity (observed: BG, NAG, PPO). Path coefficients were validated with 1,000 bootstraps, and model fit was evaluated using the Goodness-of-Fit (GoF) index, where GoF > 0.6 indicated excellent fit for soil-microbe systems (Rillig et al., 2017).

## 3 Results

### 3.1 Soil physicochemical properties

This study systematically investigated the effects of the SR-OS2 on the physicochemical properties of continuous cropping soil. The results (Table 1) demonstrated that the SMS application (B2) significantly increased soil pH and reduced EC, indicating a trend toward mitigating soil acidification and salinization, even though the initial pH was near neutral. A significant increase in soil pH of 0.57 units (*p* < 0.05) and a 37.56% reduction in EC. This improvement created a more favorable soil environment for subsequent crop growth. Regarding soil nutrients, after the first sunflower crop (B3), SOC and TN contents increased significantly by 20.6 and 23.0%, respectively, compared to the control (B1) (*p* < 0.05), while AP content increased significantly by 48.1% (*p* < 0.05). Following the harvest of the second sunflower crop (B4), SOC and TN contents remained at elevated levels, and AP content further increased to 111.16 mg/kg, representing a significant 84.2% increase over the control. These results indicate that the combination of SMS application and ornamental sunflower cultivation significantly promoted the accumulation of SOC and nitrogen, and enhanced phosphorus availability. Correlation analysis (Supplementary Table S1) revealed significant positive correlations between SOC and both TN (*r* = 0.66*) and AP (*r* = 0.70*), suggesting that increased SOC contributes to nitrogen and phosphorus accumulation. Concurrently, a highly significant negative correlation was observed between EC and pH (*r* = −0.87**), indicating that the SMS application effectively mitigated soil acidification by reducing EC.

**Table 1:** Comparison of soil physicochemical properties under different treatments.

Indicator	B1	B2	B3	B4
pH	7.68 ± 0.13 b	8.05 ± 0.08 a	7.62 ± 0.15 b	7.72 ± 0.06 b
EC (μS/cm)	131.33 ± 3.27 a	82.00 ± 4.58 b	135.00 ± 10.25 a	128.00 ± 6.08 a
SOC (%)	1.16 ± 0.06 b	1.11 ± 0.07 b	1.40 ± 0.06 a	1.39 ± 0.08 a
TN (%)	0.13 ± 0.01 b	0.13 ± 0.01 b	0.16 ± 0.01 a	0.16 ± 0.01 a
AP (mg/kg)	60.34 ± 3.23 c	63.42 ± 1.49 c	89.41 ± 5.15 b	111.16 ± 2.67 a
AK (mg/kg)	390.52 ± 9.83 b	450.91 ± 6.23 a	486.55 ± 35.46 a	372.41 ± 27.96 b

Data are mean ± standard deviation (*n* = 3); Different lowercase letters within a row indicate significant differences among treatments (*p* < 0.05, LSD test); pH, potential of hydrogen; EC, Electrical Conductivity; SOC, soil organic carbon; TN, total nitrogen; AP, available phosphorus; AK, Available Potassium.

**Figure 1:**
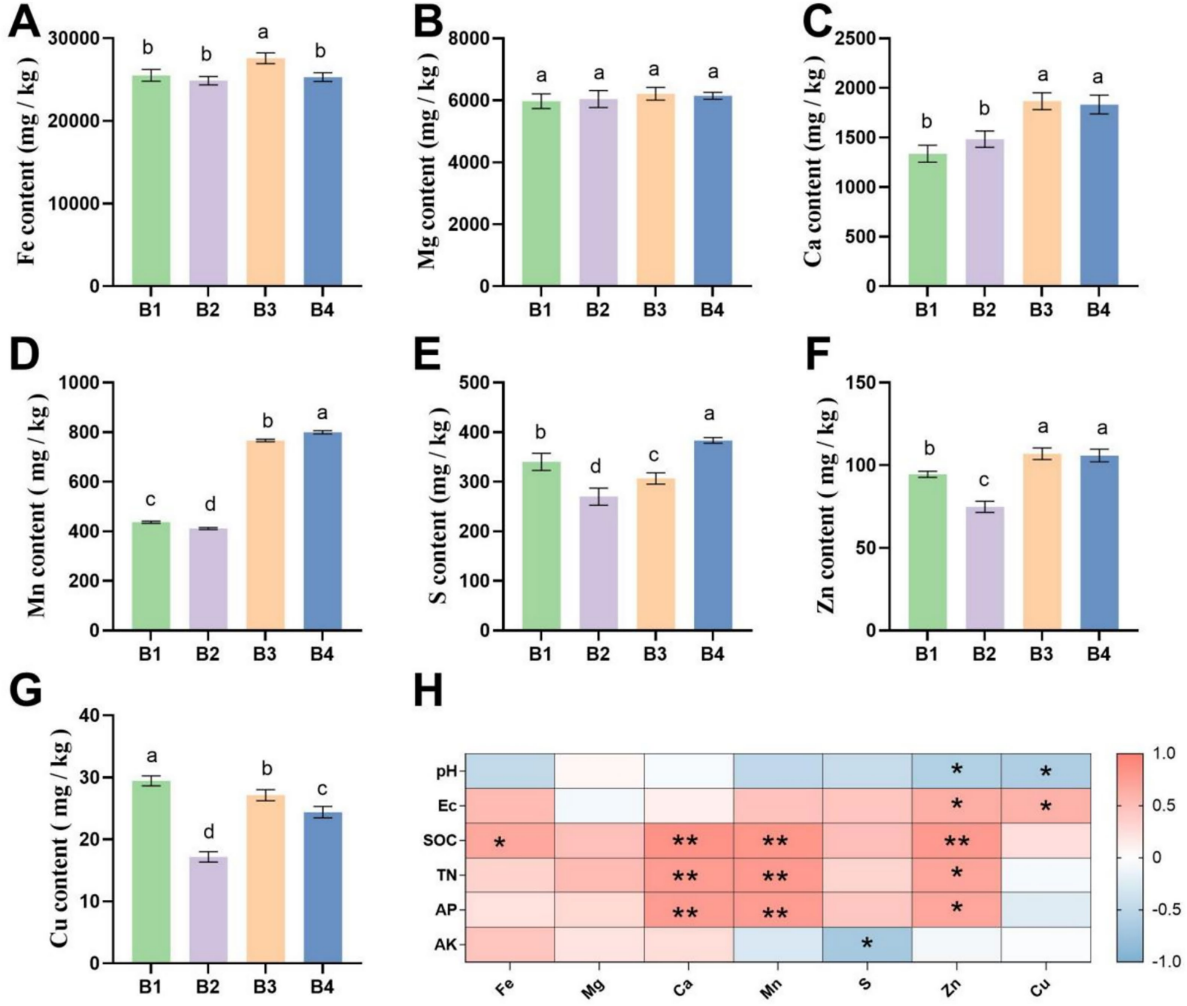
Differences in soil meso- and micro-element contents under SR-OS2 system treatments. **(A–G)** Contents of Fe, Mg, Ca, Mn, S, Zn, and Cu. Different lowercase letters indicate significant differences among treatments (*p* < 0.05, LSD test). **(H)** Correlation between soil basic nutrients and meso−/micro-elements. * denotes *p* < 0.05; ** denotes *p* < 0.01; Fe, Iron; Mg, Magnesium; Ca, Calcium; Mn, Manganese; S, Sulfur; Zn, Zinc; Cu, Copper; pH, potential of hydrogen; EC, Electrical Conductivity; SOC, soil organic carbon; TN, total nitrogen; AP, available phosphorus; AK, Available Potassium.

Figure 1 illustrates the dynamics of soil trace element contents. B2 had no significant effect on Fe, Mg, or Ca contents. However, under B3 and B4 treatments, Ca content increased significantly by 26 and 24%, respectively, compared to B2 (*p* < 0.05, Figures 1A–C). Mn, S, Zn, and Cu contents decreased after SMS application but rebounded significantly following sunflower cultivation (Figures 1D–G). Notably, in the B4 treatment, Mn, S, and Zn contents were significantly higher than in B1, with increases of 83, 13, and 12%, respectively (*p* < 0.05). Further correlation analysis indicated positive relationships of Ca and Zn with SOC and TN, while Cu showed a negative correlation with EC (Figure 1H). This suggests that the rotation system significantly influenced the availability of meso- and micro-elements by regulating organic matter decomposition.

In conclusion, the SR-OS2 system, through the synergistic effect of SMS application and sunflower cultivation, significantly improved the physicochemical properties of continuous cropping soil, thereby creating more favorable conditions for crop growth. These findings provide an important theoretical basis for utilizing agricultural waste like SMS to improve soil quality and promote the sustainable development of protected agriculture.

### 3.2 Changes in extracellular enzyme activities and stoichiometric ratios

This study comprehensively analyzed the effects of the SR-OS2 on soil extracellular enzyme activities and their stoichiometric ratios. The results demonstrated significant treatment-dependent variations in soil enzyme activities. As shown in Table 2, the SMS application (B2) increased the activities of the nitrogen (N)-cycling enzyme LAP and phosphorus (P)-cycling enzyme ALP by 42.1 and 46.0%, respectively (*p* < 0.05), while reducing BG activity (C-cycling) by 17.2% (*p* < 0.05). This shift indicates enhanced microbial demand for N and P acquisition following SMS amendment. After sunflower cultivation (B3), PPO activity peaked at 67.8% above the control (B1) (*p* < 0.05), suggesting accelerated plant residue decomposition. By the second crop (B4), LAP activity further increased by 103.1% compared to B1, whereas NAG activity remained unchanged. This reflects persistent N-cycling enhancement, potentially constrained by fungal metabolism, since NAG is primarily fungal-derived, while LAP is bacterial-dominated (Xing et al., 2024).

**Table 2:** Changes in soil extracellular enzyme activities under different treatments.

Enzyme (μmol/d/g)	B1	B2	B3	B4
BG	62.74 ± 4.72 a	51.90 ± 4.01 b	44.87 ± 3.38 c	52.58 ± 0.57 b
NAG	20.94 ± 1.18 b	21.96 ± 1.34 b	25.45 ± 0.89 a	21.06 ± 0.31 b
LAP	10.50 ± 0.51 d	14.92 ± 0.21 c	29.37 ± 0.78 a	21.33 ± 0.89 b
ALP	5.91 ± 0.33 c	8.63 ± 0.36 a	6.95 ± 0.45 b	6.75 ± 0.10 b
PPO	32.17 ± 2.06 d	36.35 ± 2.07 c	53.98 ± 2.03 a	44.56 ± 1.95 b

Data are mean ± standard deviation (*n* = 3); Different lowercase letters within a row indicate significant differences among treatments (*p* < 0.05, LSD test); BG, β-glucosidase; NAG, N-acetylglucosaminidase; LAP, leucine aminopeptidase; PPO, polyphenol oxidase.

**Figure 2:**
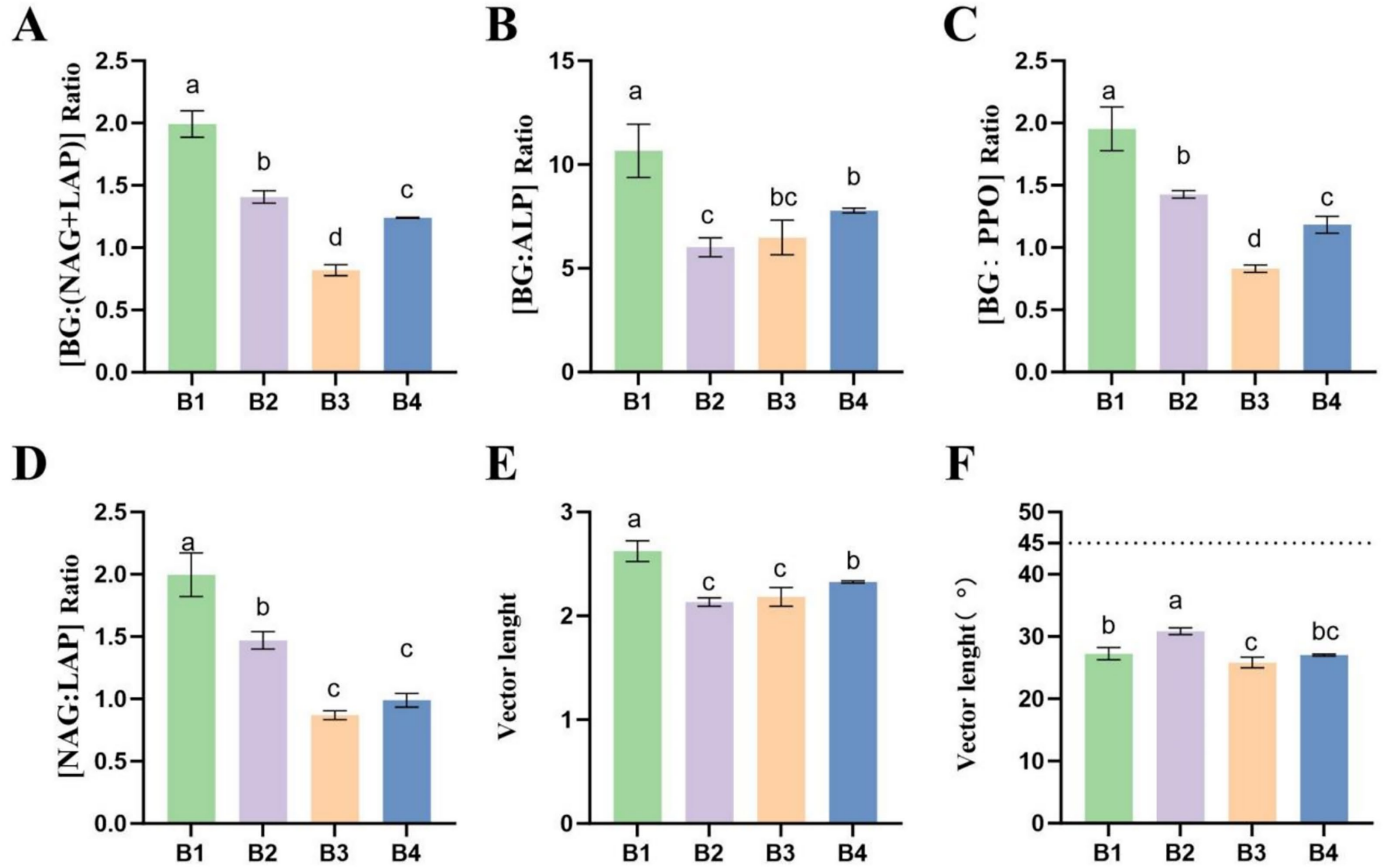
Soil enzyme stoichiometric indices under the SR-OS2 system; **(A)** BG:(NAG + LAP); **(B)** BG:ALP; **(C)** BG:PPO; **(D)** NAG:LAP; **(E)** Vector length; **(F)** Vector angle; BG, *β*-glucosidase; NAG, N-acetylglucosaminidase; LAP, leucine aminopeptidase; PPO, polyphenol oxidase.

Enzyme stoichiometric ratio analysis (Figure 2) further elucidated microbial nutrient acquisition strategies. The SMS application significantly reduced the BG:(NAG + LAP) ratio, indicating that the increase in N-cycling enzymes surpassed that of C-cycling BG (Figure 2A), thus relatively enhancing soil N-cycling capacity. Concurrently, decreased BG:ALP and BG:PPO ratios suggested improved P-cycling efficiency (Figures 2B,C). Shifts in the NAG:LAP ratio revealed altered fungal versus bacterial contributions to N-cycling (Figure 2D). Ecological enzyme stoichiometry vector analysis showed that SMS amendment significantly decreased vector length (Vector L), indicating alleviated microbial C limitation. The vector angle (Vector A) increased from 27.2° to 30.9° (remaining <45°) (Figures 2E,F), demonstrating mitigated yet persistent N limitation.

In summary, the SR-OS2 system significantly modulated soil enzyme activities and stoichiometry through SMS amendment and sunflower cultivation. The SMS application stimulated microbial demand for N and P, elevating corresponding enzyme activities, while sunflower cultivation further intensified N-cycling enzyme activity. These dynamic responses reflect microbial adaptations to soil nutrient alterations, providing critical insights into the mechanisms through which rotation systems regulate soil microecology.

### 3.3 Soil microbial diversity and community structure

**Figure 3:**
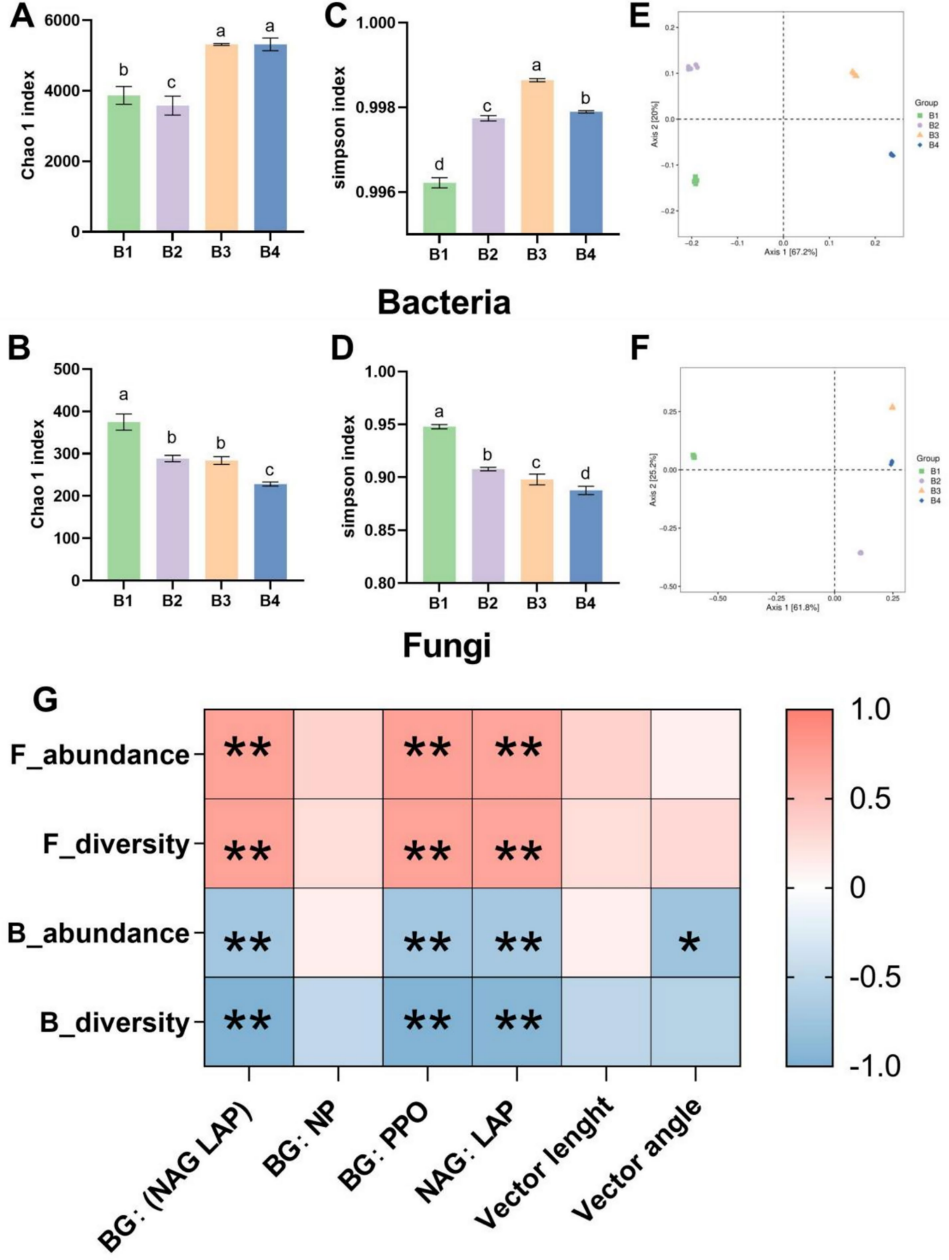
Microbial community abundance, diversity, and enzyme activity correlations under the SR-OS2 system; **(A)** Bacterial Chao1 index; **(B)** Bacterial Simpson index; **(C)** Fungal Chao1 index; **(D)** Fungal Simpson index; **(E)** Principal Coordinate Analysis (PCoA) of bacterial communities; **(F)** PCoA of fungal communities; **(G)** Correlation heatmap between microbial diversity indices and enzyme activities. Different lowercase letters indicate significant differences among treatments (*p* < 0.05, ANOVA with LSD test). * denotes *p* < 0.05; F_abundance, fungal abundance; F_diversity, fungal diversity; B_abundance, bacterial abundance; B_diversity, bacterial diversity; BG, β-glucosidase; NAG, N-acetylglucosaminidase; LAP, leucine aminopeptidase; PPO, polyphenol oxidase.

The effects of the SR-OS on soil microbial diversity and community structure were analyzed using high-throughput sequencing. The results revealed significant alterations in bacterial and fungal diversity and composition across treatments. As shown in Figures 3A,B, the bacterial Chao1 index decreased by 7.5% (*p* < 0.05) in the SMS-amended treatment (B2) compared to the control (B1). However, it increased significantly by 37.3 and 37.4% (*p* < 0.05) following the first (B3) and second sunflower crops (B4), respectively. Conversely, the fungal Chao1 index declined by 23.0% (*p* < 0.05) in B2 and decreased progressively to 39.1% below B1 (*p* < 0.05) by B4 (Figure 3B). Simpson index analysis (Figures 3C,D) indicated peak bacterial diversity in B3, while fungal diversity in B2 was significantly lower than in B1 (*p* < 0.05). This demonstrates that the rotation system enhanced bacterial diversity and evenness but suppressed fungal diversity and evenness post-sunflower cultivation.

Principal Coordinates Analysis (PCoA) based on Bray-Curtis distances revealed significant divergence in microbial community composition among treatments. Bacterial PCoA (Figure 3E) showed clear separation between B1/B2 and B3/B4 clusters along the first principal component, indicating that SMS amendment and sunflower cultivation substantially restructured bacterial communities. Fungal PCoA (Figure 3F) exhibited similar clustering patterns, confirming treatment-dependent shifts in microbial assemblages.

Correlation analysis (Figure 3G) identified significant associations between bacterial diversity and enzyme stoichiometric ratios. Increased BG:(NAG+LAP), BG:PPO, and NAG:LAP ratios correlated with enhanced fungal diversity/richness but reduced bacterial diversity/richness (*p* ≤ 0.05). Additionally, negative correlations between bacterial abundance and vector angles underscored linkages between community structure and enzymatic activity dynamics.

In summary, the SR-OS2 system significantly reshaped soil microbial diversity and community structure through SMS amendment and sunflower cultivation. Initial suppression of fungal diversity was followed by robust bacterial diversification during rotation progression, demonstrating the system’s capacity to restore bacterial communities. These shifts provide critical insights into the mechanisms through which rotation systems regulate soil microecology.

### 3.4 Impact of the rotation system on soil microbial co-occurrence networks

**Figure 4:**
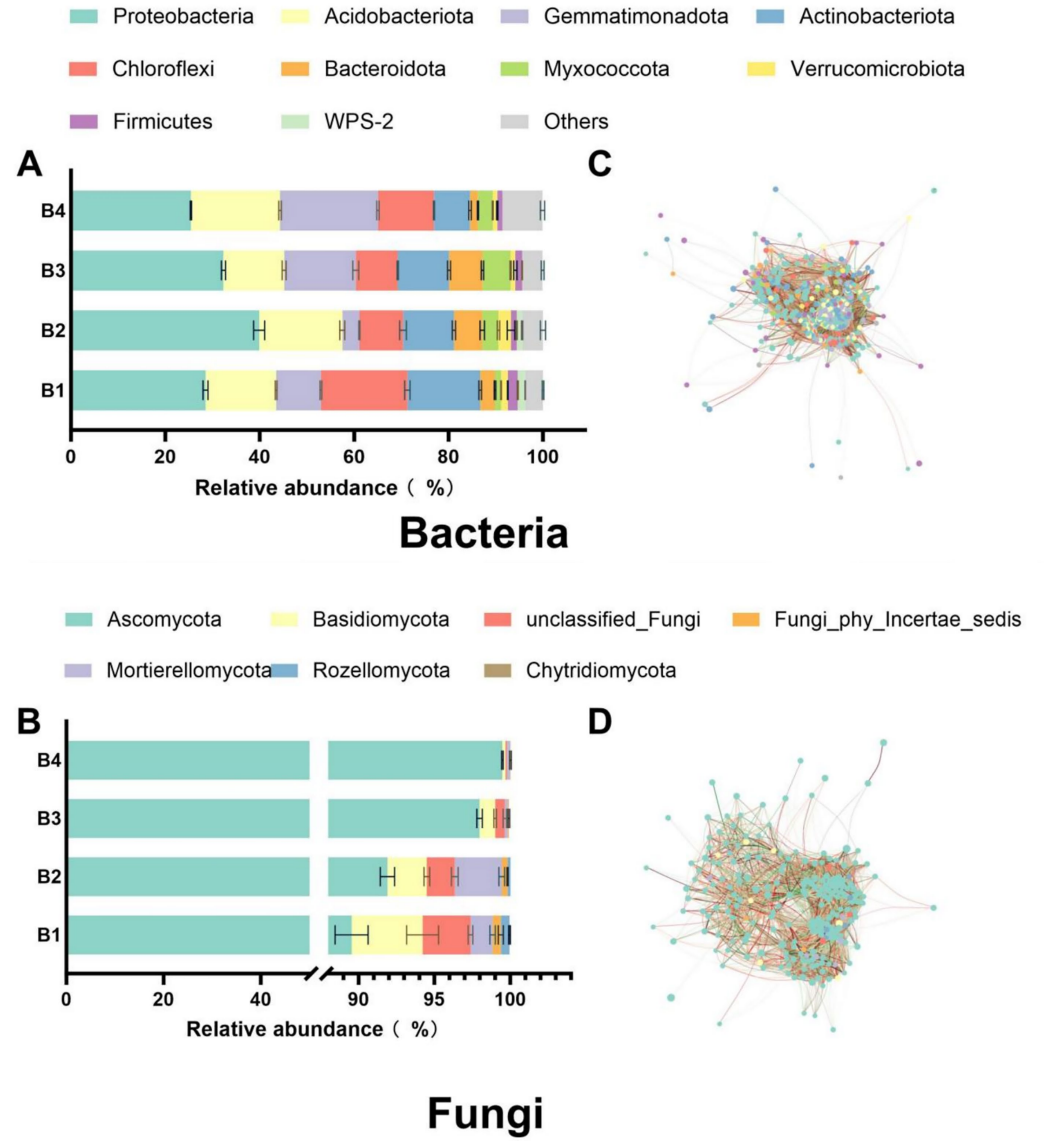
Phylogenetic profiling and co-occurrence network analysis of dominant bacterial and fungal phyla under the SR-OS2 system; **(A)** Relative abundance of top 10 bacterial phyla; **(B)** Relative abundance of dominant fungal phyla; **(C)** Co-occurrence network of top 10 bacterial phyla; **(D)** Co-occurrence network of dominant fungal phyla. Network edges represent significant species correlations (*p* < 0.05), with line thickness proportional to correlation coefficient (r). Red lines: positive correlations; Green lines: negative correlations. Node size reflects connectivity degree.

The SR-OS2 significantly restructured soil microbial community composition and co-occurrence networks through SMS amendment and sunflower cultivation. In bacterial communities, the SMS application (B2) markedly increased the relative abundance of *Proteobacteria* and *Acidobacteriota* while reducing *Chloroflexi*, *Actinobacteriota*, and *Gemmatimonadota*. Subsequent sunflower cultivation (B3/B4) induced a progressive decline in *Proteobacteria*, with *Acidobacteriota* and *Chloroflexi* showing initial reduction followed by rebound, while *Gemmatimonadota* demonstrated an increasing trend (Figure 4A). For fungi, *Ascomycota* remained dominant, with SMS amendment elevating *Ascomycota* and *Mortierellomycota* but suppressing *Rozellomycota*, *Basidiomycota*, and *Chytridiomycota*. By B3/B4, *Ascomycota* increased by 9.4 and 11.1% versus B1 (*p* < 0.05), contrasting with drastic declines in *Basidiomycota* (−78%/−96%), *Mortierellomycota* (−87%/−88%), *Rozellomycota* (−95%/−100%), and *Chytridiomycota* (−100%/−100%) (Figure 4C).

Network topology analysis revealed that SMS amendment and sunflower cultivation substantially modified microbial interactions. Bacterial networks exhibited enhanced scale and connectivity in B2-B4, evidenced by increased node counts and average degrees, with peak complexity in B3 despite slightly reduced transitivity (Figure 4B). Fungal networks showed strengthened diversity and interactions under SMS (B2), but no-till sunflower cultivation (B3/B4) reduced node counts, links, and average degrees, indicating diminished connectivity (Figure 4D). Collectively, these findings demonstrate that SMS amendment enhanced network complexity in both domains, while subsequent sunflower cultivation exerted divergent effects, further complexifying bacterial networks but simplifying fungal networks. This restructuring provides critical insights for designing soil management strategies to optimize ecological functioning.

### 3.5 Cluster and redundancy analyses integrating environmental factors and microbial communities

**Figure 5:**
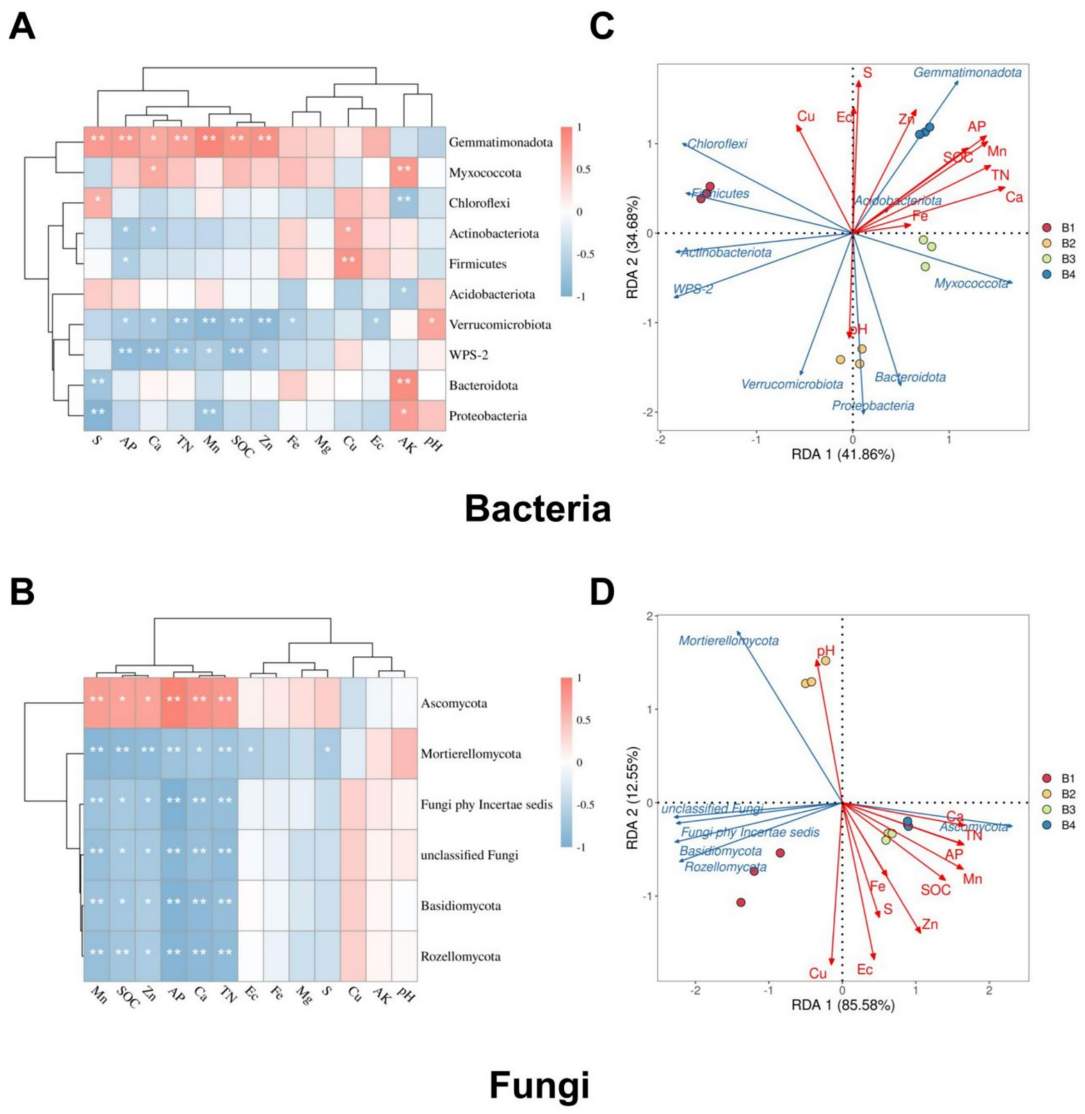
Integrated cluster and redundancy analyses of soil microbial phyla with environmental factors under the SR-OS2 system; **(A)** Cluster analysis of bacterial phyla based on soil physicochemical properties; **(B)** Cluster analysis of fungal phyla based on soil physicochemical properties; **(C)** Redundancy analysis (RDA) of bacterial phyla-environmental relationships (RDA1 = 41.86%, RDA2 = 34.68%); **(D)** RDA of fungal phyla-environmental relationships (RDA1 = 85.58%, RDA2 = 12.55%). Green arrows represent microbial taxa; Solid red arrows represent soil physicochemical properties; Fe, Iron; Mg, Magnesium; Ca, Calcium; Mn, Manganese; S, Sulfur; Zn, Zinc; Cu, Copper; pH, potential of hydrogen; EC, Electrical Conductivity; SOC, soil organic carbon; TN, total nitrogen; AP, available phosphorus; AK, Available Potassium.

Cluster analysis and RDA, integrating environmental factors and microbial data, further elucidated the regulatory mechanisms of soil physicochemical properties on microbial community structure. Cluster analysis based on bacterial (Figure 5A) and fungal abundance (Figure 5B) with soil properties revealed phylum-specific responses to environmental factors. *Proteobacteria* were inhibited by TN, preferring low-N environments. In contrast, *Gemmatimonadota* showed significant positive correlations with S, Ca, Mn, Zn, TN, and soil SOC, indicating a competitive advantage in nutrient-rich soils. *Actinobacteriota* exhibited negative correlations with AK and pH, thriving in acidic, low-K conditions. *Bacteroidota* dominated in low-S/high-P soils, negatively correlating with S but positively with AP. These results demonstrate that soil physicochemical properties fundamentally shape microbial community assembly.

RDA quantified environmental drivers of community variation. For bacteria, RDA1 and RDA2 explained 41.86 and 34.68% of abundance variation (cumulative 76.54%; Figure 5C). *Actinobacteriota* and *Proteobacteria* positively correlated with SOC, TN, and Ca, highlighting their competitiveness in organically enriched soils. Conversely, *Acidobacteriota* and *Gemmatimonadota* showed negative correlations with S, EC, Zn, AP, and Cu, indicating a preference for low-S, low-EC, and low-Zn environments. For fungi, RDA1 captured 85.58% of the variation with RDA2 explaining 12.55% (cumulative 98.13%; Figure 5D). *Ascomycota* and *Basidiomycota* are positively associated with TN and Ca, dominating high-N/high-Ca soils. *Mortierellomycota* are closely linked with SOC, iron (Fe), and Mn, while unclassified fungi and *Rozellomycota* were negatively regulated by S, Zn, EC, Cu, and AP, indicating competitiveness in low-S, low-Zn, low-EC, low-Cu, and low-P environments.

Collectively, these analyses demonstrate that key elements (N, Ca, S, Zn, Cu) critically influence microbial distribution patterns through physicochemical regulation of community structure, providing fundamental insights into soil microecological functionality and its governing mechanisms.

### 3.6 Microbial succession and functional shifts

The SR-OS2 system induced a stage-specific succession of soil microbial communities. LEfSe analysis showed that the untreated soil (B1) was dominated by generalist decomposers (e.g., *Sphingomonas*, *Penicillium*). SMS amendment (B2) enriched specialized decomposers, including the bacteria *Dyella* and *Rhodanobacter*, and the nematode-trapping fungus *Arthrobotrys*. Sunflower cultivation (B3/B4) further shifted the community toward nutrient-mobilizing and antipathogenic taxa, such as the phosphate solubilizer *Gemmatimonas* and the antipathogenic bacterium *Lysobacter* (Supplementary Table S3).

FUNGuild analysis revealed a functional transition in fungal trophic modes. From B1 to B4, the pure saprotrophic and parasitic-saprotrophic-symbiotic guilds increased dramatically (22.2-fold and 9.1-fold, respectively), while the parasitic-saprotrophic guild decreased (Supplementary Table S4). Compared with B1, the relative abundance of potential fungal pathogens (e.g., *Fusarium*) was significantly reduced in B3 and B4 treatments. This indicates a systematic shift toward saprotrophy and multifunctionality, reducing pathogen pressure.

### 3.7 Partial least squares path modeling (PLS-PM)

**Figure 6:**
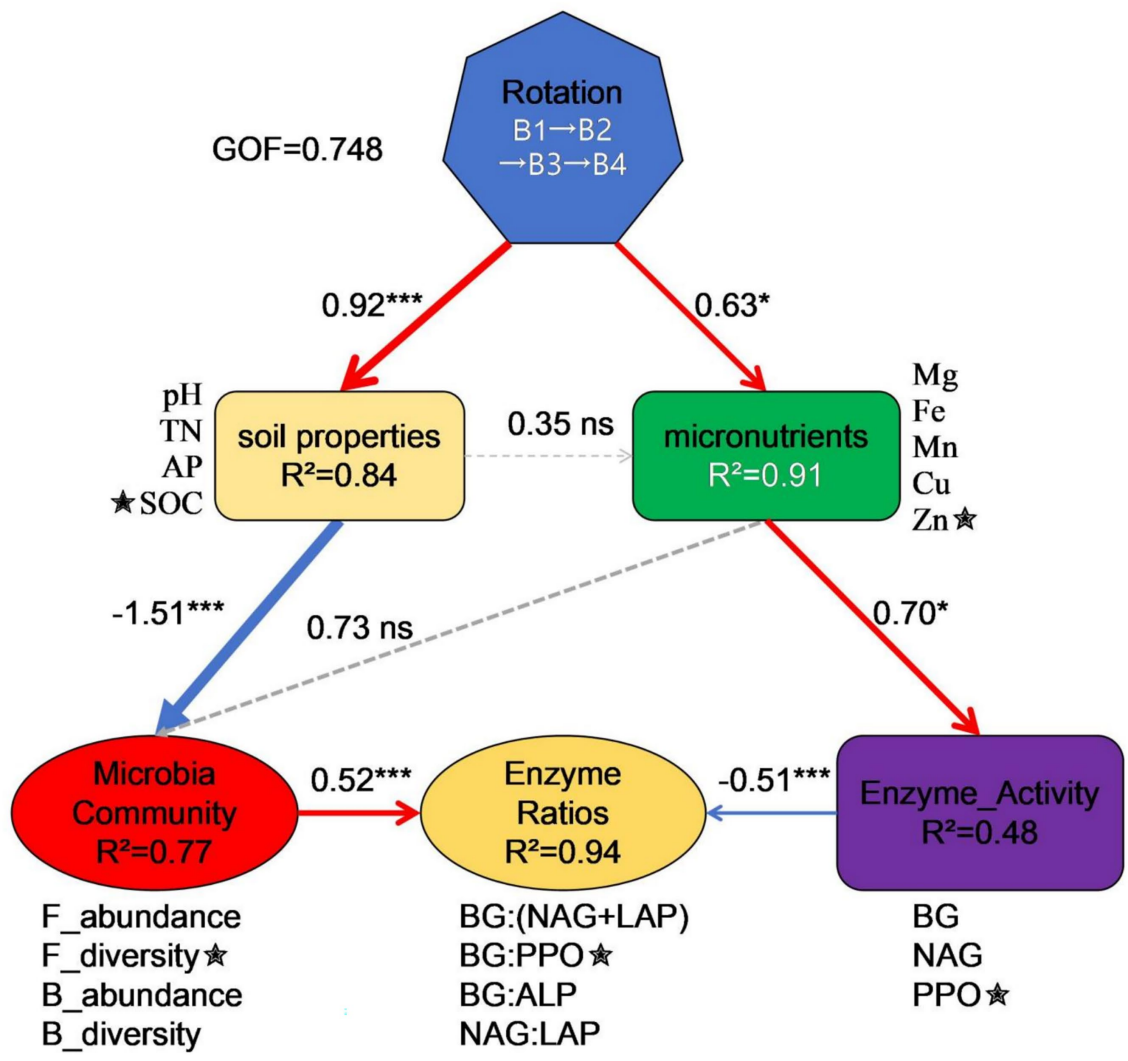
PLS-PM of microecological restoration mechanisms in the SR-OS2 rotation system, Solid red lines: Positive paths, Solid blue lines: Negative paths, Dashed lines: Non-significant paths, Numerical values: Path coefficients, ★: Strongest loadings, ***p < 0.001; *p < 0.05; ns *p* > 0.05, R^2^ indicates model explanatory power; GoF = 0.748; pH, potential of hydrogen; SOC, soil organic carbon; TN, total nitrogen; AP, available phosphorus; Fe, Iron; Mg, Magnesium; Mn, Manganese; Zn, Zinc; Cu, Copper; F_abundance, fungal abundance; F_diversity, fungal diversity; B_abundance, bacterial abundance; B_diversity, bacterial diversity; BG, β-glucosidase; NAG, N-acetylglucosaminidase; LAP, leucine aminopeptidase; PPO, polyphenol oxidase.

Figure 6 presents the PLS-PM elucidating the hierarchical regulatory mechanisms through which the SR-OS2 restores soil microecological functions. The model demonstrated good overall fit (GoF = 0.748), with all latent variables exhibiting average variance extracted (AVE) > 0.7, validating measurement model reliability. Two primary pathways mediated the system’s effects: (1) The *biological pathway* (Rotation → Soil properties → Microbial Community → Enzyme Ratios) showed rotation treatments (B1-B4) strongly driving soil physicochemical alterations (*β* = 0.92, *p* < 0.001; *R*^2^ = 0.84). Elevated SOC and TN in B3/B4 versus B1/B2 confirmed that SMS amendment, coupled with sunflower cultivation, enhanced soil C-N levels. These changes reduced fungal abundance in high-SOC treatments (B3: 286.6 vs. B1: 361.9), likely through carbon-triggered microbial metabolic compensatory mechanisms. Ultimately, bacterial diversity positively regulated enzyme stoichiometric ratios (*β* = 0.53, *p* = 0.003), evidenced by higher BG:PPO ratios in high-diversity B2 (1.42) versus B3 (0.80). (2) The *physicochemical pathway* (Rotation → Micronutrients → Enzyme Activity → Enzyme Ratios) revealed micronutrient-mediated enzyme activity shifts, where increased BG and PPO activities significantly reduced enzyme balance ratios (*β* = −0.501, *p* = 0.004). This was manifested by 45% greater PPO activity in B4 (45.9 μmol g^−1^ soil d^−1^) than in B1 (31.6 μmol g^−1^ soil d^−1^), alongside decreased BG:(NAG+LAP) from 1.88 to 1.24. Collectively, PLS-PM uncovered how rotation systems cascade through soil-microbe-enzyme hierarchies to restore ecological functions, providing mechanistic foundations for optimizing agricultural management.

## 4 Discussion

### 4.1 Remediation effects of SR-OS2 on physicochemical properties of continuous cropping soil

This study demonstrates that the SR-OS2 significantly ameliorates physicochemical degradation in continuous cropping soils. SMS application (B2) substantially increased soil pH while reducing EC, effectively mitigating soil acidification and salinization. These findings align with Hu et al. (2020), who reported similar pH improvements through edible mushroom residue amendment (Hu et al., 2020). The pH elevation may be attributed to carbonic anhydrase in SMS catalyzing CO₂ hydration to HCO₃^−^, thereby buffering soil acidity (Occhipinti and Boron, 2019), while SMS’s porous structure likely immobilized free salt ions through physical adsorption (Wu et al., 2019). Regarding soil nutrients, sunflower cultivation (B3/B4) significantly enhanced SOC, TN, and AP contents. SOC accumulation likely resulted from covalent bonding of lignocellulosic components forming stable organo-mineral complexes (Kleber et al., 2021) and microbial-mediated ammonification of fungal mycelia-derived amino sugars, elevating TN (Li T. et al., 2022). AP increase may stem from SMS phosphorus mineralization and phosphate-solubilizing rhizobacteria secreting acid phosphatases (Tao and Gao, 2023). Trace element dynamics revealed Ca^2+^ and Zn^2+^ enrichment via organic acid-mediated mineral weathering (Schmalenberger et al., 2015), contrasting with Cu^2+^ reduction potentially involving humus chelation (Qin et al., 2023). Significant positive correlations between SOC and both TN and AP suggest SOC accumulation facilitates nutrient retention, implying enhanced SOC inputs can improve soil fertility (Abay et al., 2024). Collectively, SR-OS2 synergistically enhanced soil properties through SMS amendment and sunflower cultivation, providing critical foundations for utilizing agricultural waste in sustainable protected agriculture.

### 4.2 Microbial nutrient acquisition strategies revealed by extracellular enzyme activity dynamics

The SR-OS2 significantly modulated soil extracellular enzyme activities and stoichiometric ratios, revealing microbial adaptive strategies to nutrient availability shifts. Following SMS amendment (B2), LAP (nitrogen-cycling) and ALP (phosphorus-cycling) activities increased substantially while BG (carbon-cycling) activity decreased. This prioritization of N and P acquisition enzymes under nutrient constraints reflects microbial optimization of substrate capture efficiency, consistent with Sinsabaugh et al.’s framework of nutrient-dependent enzyme allocation (Sinsabaugh et al., 2009). Post-sunflower cultivation (B3), peak polyphenol oxidase (PPO) activity indicated accelerated plant residue decomposition and altered carbon utilization strategies. Sunflower root exudates rich in lignin and cellulose likely stimulated microbial production of ligninolytic enzymes, driving priming effects on recalcitrant carbon decomposition as documented by Baldrian (2008) (Baldrian, 2008). SMS amendment significantly reduced the BG:(NAG+LAP) ratio, demonstrating enhanced nitrogen-cycling capacity relative to carbon acquisition, while decreased BG:ALP and BG:PPO ratios suggested improved phosphorus-cycling efficiency. Vector analysis showed reduced vector length (indicating alleviated carbon limitation) and increased vector angle from 27.2° to 30.9° (persistent nitrogen limitation despite mitigation), aligning with Moorhead’s ecological stoichiometry principles (Moorhead et al., 2016). Collectively, these enzyme dynamics demonstrate how SR-OS2 triggers microbial metabolic reprogramming in response to nutrient shifts, providing a scientific basis for optimizing agricultural management through rotation systems.

### 4.3 Microbial community restructuring and alleviation mechanisms for continuous cropping obstacles

SMS amendment induced microbial community restructuring characterized by bacterial dominance: Bacterial *α*-diversity increased progressively during rotation, while fungal diversity declined significantly. This divergence is consistent with the observed increase in oligotrophic taxa such as *Acidobacteriota* and *Chloroflexi* (Figure 4A), which are known for their ability to degrade complex aromatic compounds present in SMS (Fierer et al., 2007). Co-occurrence network analysis revealed decreased modularity but increased connectivity density in bacterial networks, indicating SMS input enhanced functional guild cooperation (Banerjee et al., 2016). Conversely, reduced keystone species in fungal networks suggest SMS effectively disrupted pathogenic niches.

Collectively, the SR-OS2 system restructured soil microbial diversity and composition through SMS amendment and sunflower cultivation. Initial fungal suppression was succeeded by significant bacterial diversification during rotation progression, demonstrating the system’s capacity to restore bacterial communities. These shifts elucidate rotation-mediated regulatory mechanisms in soil microecology and provide scientific foundations for optimizing agricultural management.

### 4.4 Microbial succession as a driver of soil restoration

The SR-OS2 system drove a deterministic microbial succession that directly remediated continuous cropping obstacles. The initial community of generalist decomposers (B1) transitioned to substrate-activated specialists (B2), initiating the breakdown of complex organics and biocontrol of nematodes. Subsequent sunflower cultivation (B3/B4) enriched taxa critical for nutrient mobilization (e.g., *Gemmatimonas*) and pathogen suppression (e.g., *Lysobacter*), establishing a resilient soil state.

The concomitant functional shift in fungi—from pathotrophic to saprotrophic dominance—reflects a systematic reduction in pathogen pressure. Thus, the system’s efficacy stems from its ability to orchestrate a phased functional succession within the microbiome, sequentially activating processes that restore soil health.

### 4.5 Driving effects of nutrient limitation on microbial communities and regulatory roles of the rotation system

Partial Least Squares Path Modeling (PLS-PM; GoF = 0.748) revealed dual cascading pathways through which the *Stropharia rugosoannulata*-Ornamental Sunflower Rotation System (SR-OS2) restores soil microecological function. In the biological pathway, SMS amendment significantly elevated soil carbon and nitrogen levels (SOC/TN, *β* = 0.92, *p* < 0.001; R^2^ = 0.84), consistent with organic amendments’ role in soil fertility reconstruction (Lal, 2004). High carbon inputs triggered microbial metabolic compensatory mechanisms (Fontaine et al., 2003), suppressing fungal abundance by 20.8% in B3 versus B1, while bacterial diversity (B_diversity) positively regulated enzyme balance (*β* = 0.53, *p* = 0.003) via functional redundancy (Wagg et al., 2019). In the physicochemical pathway, rotation altered micronutrient bioavailability, activating metalloenzyme-dependent polyphenol oxidase (PPO) (45% activity increase in B4 vs. B1) (Sinsabaugh, 2010). Coordinated enhancement of β-glucosidase (BG) and PPO activities induced microbial resource allocation trade-offs (Moorhead et al., 2016), significantly reducing enzyme balance ratios (*β* = −0.501, *p* = 0.004) and shifting soil organic matter transformation toward recalcitrant carbon decomposition (Cotrufo et al., 2013). Collectively, SR-OS2 achieves waste valorization and fertility enhancement through these synergistic pathways, with enzyme activity ratios (e.g., BG:PPO) serving as sensitive biological indicators for optimizing rotation design.

It is important to note that the primary objective of this study was to decipher the soil-mediated mechanisms (physicochemical and microecological) through which the SR-OS2 system alleviates continuous cropping obstacles. Therefore, we focused our experimental design and resources on intensive soil sampling and analysis. While plant productivity and health are the ultimate agronomic goals, they are lagging indicators that result from the complex interplay of soil conditions remediated by our system. The significant improvements in soil health parameters reported here—such as the alleviation of nutrient limitations, the restructuring of the microbial community away from pathogenic dominance, and the enhancement of beneficial bacterial networks—are well-established precursors to improved plant growth and yield. Future studies will explicitly measure the transgenerational effects of these soil improvements on sunflower agronomic traits to provide a complete assessment of the SR-OS2 system’s benefits.

## 5 Conclusion

The SR-OS2 system synergistically improved physicochemical properties of continuous cropping soils through integrated SMS application and rotational cultivation. It enhanced nitrogen/phosphorus-cycling enzyme activities, restructured microbial communities, effectively alleviated nutrient limitations, and suppressed pathogen enrichment. These findings establish a technical framework for ecologically controlling sunflower continuous cropping obstacles while providing novel approaches for agricultural waste valorization and sustainable protected agriculture. Future research should elucidate mechanisms of functional microorganisms in SMS, optimize rotation cycles and amendment rates, and advance precision management of this system.

## Data Availability

The data of this study have been deposited in the Genome Sequence Archive database, and the accession number is PRJCA049688.

## Footnotes

^1^ https://huttenhower.sph.harvard.edu/galaxy/
