# Supplementary material for: Synergistic remediation of continuous cropping obstacles in facility agriculture: insights from the Stropharia rugosoannulata-Ornamental Sunflower Rotation System
Source: Front Microbiol. 2025 Nov 7;16:1671484. doi: 10.3389/fmicb.2025.1671484 (PMC12634544; doi:10.3389/fmicb.2025.1671484)
Supplement: Supplementary file 1 [file Table_1.DOCX]

Table S1: Pearson correlation coefficients between soil basic nutrients and meso-/micro-elements under SR-OS2 treatments

|  | NT | EC | pH | AP | Mg | S | Ca | Fe | Mn | Cu | Zn | SOC | AK |
| --- | --- | --- | --- | --- | --- | --- | --- | --- | --- | --- | --- | --- | --- |
| NT | 1.00 |  |  |  |  |  |  |  |  |  |  |  |  |
| EC | 0.11 | 1.00 |  |  |  |  |  |  |  |  |  |  |  |
| pH | -0.22 | -0.87** | 1.00 |  |  |  |  |  |  |  |  |  |  |
| AP | 0.86** | 0.06 | -0.04 | 1.00 |  |  |  |  |  |  |  |  |  |
| Mg | 0.52 | -0.09 | 0.05 | 0.29 | 1.00 |  |  |  |  |  |  |  |  |
| S | 0.33 | 0.44 | -0.44 | 0.44 | 0.16 | 1.00 |  |  |  |  |  |  |  |
| Ca | 0.77** | 0.11 | -0.06 | 0.78** | 0.50 | 0.23 | 1.00 |  |  |  |  |  |  |
| Fe | 0.34 | 0.53 | -0.48 | 0.23 | 0.32 | 0.05 | 0.43 | 1.00 |  |  |  |  |  |
| Mn | 0.79** | 0.47 | -0.50 | 0.77** | 0.45 | 0.77** | 0.71* | 0.34 | 1.00 |  |  |  |  |
| Cu | -0.07 | 0.61* | -0.63* | -0.25 | 0.07 | 0.38 | -0.15 | 0.51 | 0.22 | 1.00 |  |  |  |
| Zn | 0.70* | 0.64* | -0.60* | 0.69* | 0.18 | 0.57 | 0.63* | 0.56 | 0.83** | 0.27 | 1.00 |  |  |
| SOC | 0.66* | 0.46 | -0.39 | 0.70* | 0.49 | 0.50 | 0.85** | 0.69* | 0.82** | 0.25 | 0.79** | 1.00 |  |
| AK | -0.00 | -0.14 | 0.06 | -0.18 | 0.20 | -0.68* | 0.27 | 0.44 | -0.30 | -0.04 | -0.09 | 0.15 | 1.00 |

Table S2: Collinearity network picture information.

| Treatments | | Nodes | Links | Transitivity | Average path length | Diameter | Average degree |
| --- | --- | --- | --- | --- | --- | --- | --- |
| Bacteria | B1 | 855±11 d | 276627±6348 c | 0.91±0.00 a | 1.29±0.01 c | 3±0.04 b | 1872±29 d |
|  | B2 | 913±22 c | 276340±8875 c | 0.90±0.01 b | 1.44±0.02 b | 4±0.04 a | 2019±55 c |
|  | B3 | 1150±15 a | 354681±7844 b | 0.84±0.01 c | 1.58±0.01 a | 4±0.04 a | 2729±44 a |
|  | B4 | 1107±14 b | 385495±6558 a | 0.83±0.00 d | 1.43±0.01 b | 4±0.14 a | 2658±40 b |
| Fungi | B1 | 95±4 c | 1924±39 c | 0.82±0.01 a | 1.66±0.04 c | 3±0.26 a | 41±1 b |
|  | B2 | 131±2 a | 3168±143 a | 0.81±0.01b | 1.71±0.01 b | 3±0.10 a | 48±1 a |
|  | B3 | 126±2 b | 1959±86 bc | 0.71±0.01 c | 1.90±0.02 a | 3±0.23 a | 31±1 c |
|  | B4 | 127±3 ab | 2083±104 b | 0.70±0.01 c | 1.89±0.00 a | 3±0.20 a | 32±1 c |

Table S3: lefse results

| Kingdom | Taxa | Abundace | Group | LDA_score | Pvalue |
| --- | --- | --- | --- | --- | --- |
| Bacteria | Bacteria.Proteobacteria.Alphaproteobacteria.Sphingomonadales.Sphingomonadaceae.Sphingomonas | 4.96 | B1 | 4.62 | 0.0037 |
|  | Bacteria.Proteobacteria.Gammaproteobacteria.Xanthomonadales.Rhodanobacteraceae.Dyella | 5.04 | B2 | 4.74 | 0.0004 |
|  | Bacteria.Chloroflexi.Ktedonobacteria.Ktedonobacterales.JG30_KF_AS9.JG30_KF_AS9 | 4.49 | B1 | 4.12 | 0.0069 |
|  | Bacteria.Proteobacteria.Alphaproteobacteria.Reyranellales.Reyranellaceae.Reyranella | 4.32 | B3 | 4.04 | 0.0009 |
|  | Bacteria.Proteobacteria.Gammaproteobacteria.Burkholderiales.SC_I_84.SC_I_84 | 5.50 | B4 | 5.18 | 0.0006 |
|  | Bacteria.Patescibacteria.Parcubacteria.Candidatus_Jorgensenbacteria.Candidatus_Jorgensenbacteria.Candidatus_Jorgensenbacteria | 4.47 | B4 | 4.17 | 0.0010 |
|  | Bacteria.Proteobacteria.Alphaproteobacteria.Dongiales.Dongiaceae.Dongia | 4.55 | B3 | 4.22 | 0.0037 |
|  | Bacteria.Proteobacteria.Gammaproteobacteria.Burkholderiales.Burkholderiaceae.Burkholderia_Caballeronia_Paraburkholderia | 4.67 | B1 | 4.38 | 0.0009 |
|  | Bacteria.Actinobacteriota.Actinobacteria.Frankiales.Acidothermaceae.Acidothermus | 4.87 | B3 | 4.58 | 0.0010 |
|  | Bacteria.Gemmatimonadota.Gemmatimonadetes.Gemmatimonadales.Gemmatimonadaceae.Gemmatimonas | 5.12 | B3 | 4.82 | 0.0009 |
|  | Bacteria.Proteobacteria.Gammaproteobacteria.Xanthomonadales.Xanthomonadaceae.Lysobacter | 4.42 | B4 | 4.12 | 0.0005 |
|  | Bacteria.Actinobacteriota.Actinobacteria.Pseudonocardiales.Pseudonocardiaceae.Pseudonocardia | 4.55 | B3 | 4.27 | 0.0031 |
|  | Bacteria.Proteobacteria.Gammaproteobacteria.Xanthomonadales.Rhodanobacteraceae.Luteibacter | 4.38 | B1 | 4.11 | 0.0004 |
|  | Bacteria.Proteobacteria.Gammaproteobacteria.Xanthomonadales.Rhodanobacteraceae.Rhodanobacter | 5.22 | B2 | 4.90 | 0.0020 |
| Fungi | Fungi.Ascomycota.Eurotiomycetes.Eurotiales.Aspergillaceae.Penicillium | 4.78 | B1 | 4.46 | 0.0005 |
|  | Fungi.Ascomycota.Sordariomycetes.Glomerellales.Plectosphaerellaceae.Plectosphaerella | 5.33 | B1 | 5.03 | 0.0068 |
|  | Fungi.Ascomycota.Dothideomycetes.Capnodiales.Cladosporiaceae.Cladosporium | 4.82 | B1 | 4.52 | 0.0005 |
|  | Fungi.Ascomycota.Sordariomycetes.Sordariales.Chaetomiaceae.Humicola | 4.42 | B1 | 4.12 | 0.0023 |
|  | Fungi.Ascomycota.Sordariomycetes.Sordariales.Lasiosphaeriaceae.Arnium | 5.02 | B1 | 4.72 | 0.0022 |
|  | Fungi.Ascomycota.Sordariomycetes.Hypocreales.Sarocladiaceae.Parasarocladium | 4.94 | B1 | 4.64 | 0.0005 |
|  | Fungi.Basidiomycota.Agaricomycetes.Agaricales.Strophariaceae.Stropharia | 4.89 | B1 | 4.58 | 0.0006 |
|  | Fungi.Ascomycota.Leotiomycetes.Helotiales.Helotiaceae.Scytalidium | 4.70 | B2 | 4.40 | 0.0005 |
|  | Fungi.Ascomycota.Orbiliomycetes.Orbiliales.Orbiliaceae.Arthrobotrys | 4.41 | B2 | 4.11 | 0.0011 |
|  | Fungi.Ascomycota.Sordariomycetes.Chaetosphaeriales.Chaetosphaeriaceae.Chaetosphaeria | 5.64 | B2 | 5.34 | 0.0005 |
|  | Fungi.Basidiomycota.Agaricomycetes.Polyporales.Phanerochaetaceae.Phanerodontia | 4.47 | B2 | 4.18 | 0.0005 |
|  | Fungi.Ascomycota.Sordariomycetes.Hypocreales.Hypocreaceae.Monocillium | 5.44 | B2 | 5.14 | 0.0004 |
|  | Fungi.Ascomycota.Eurotiomycetes.Eurotiales.Aspergillaceae.Aspergillus | 5.26 | B3 | 4.85 | 0.0005 |
|  | Fungi.Ascomycota.Eurotiomycetes.Chaetothyriales.Herpotrichiellaceae.Phialophora | 4.87 | B3 | 4.57 | 0.0010 |
|  | Fungi.Ascomycota.Sordariomycetes.Microascales.Microascaceae.Cephalotrichum | 4.70 | B3 | 4.41 | 0.0006 |
|  | Fungi.Ascomycota.Sordariomycetes.Microascales.Microascaceae.Microascus | 4.30 | B3 | 4.02 | 0.0005 |
|  | Fungi.Ascomycota.Sordariomycetes.Hypocreales.Stachybotryaceae.Stachybotrys | 1.79 | B3 | 4.04 | 0.0304 |
|  | Fungi.Ascomycota.Sordariomycetes.Sordariales.Lasiosphaeriaceae.Cercophora | 4.41 | B4 | 4.12 | 0.0005 |
|  | Fungi.Ascomycota.Eurotiomycetes.Eurotiales.Trichocomaceae.Thermomyces | 5.60 | B4 | 5.29 | 0.0006 |

Table S4: FUNGuild analysis results

| TrophicMode | B1 | B2 | B3 | B4 | fold change（B4/B1） |
| --- | --- | --- | --- | --- | --- |
| Pathotroph-Saprotroph-Symbiotroph | 0.0473 | 0.2575 | 0.1318 | 0.4290 | 9.1 |
| Saprotroph | 0.0090 | 0.0224 | 0.0183 | 0.1995 | 22.2 |
| Pathotroph-Saprotroph | 0.0628 | 0.0272 | 0.0333 | 0.0223 | 2.8（B1/B4） |
